# Real-time multispectral imaging for intraoperative monitoring of coronary artery bypass graft patency

**DOI:** 10.1117/1.JBO.30.3.036001

**Published:** 2025-03-10

**Authors:** Jens De Winne, Danilo Babin, Hiep Luong, Siri Luthman, Aleksandar M. Milosavljević, Živojin Jonjev, Jan Hrubik, Lazar Velicki

**Affiliations:** a Ghent University, Image Processing and Interpretation, Department of Telecommunications and Information Processing, Ghent, Belgium; b Interuniversity Micro-Electronics Center (IMEC) vzw, Leuven, Belgium; c Institute for Cardiovascular Diseases of Vojvodina, Sremska Kamenica, Serbia; d University of Novi Sad, Faculty of Medicine, Novi Sad, Serbia; e University of Banja Luka, Faculty of Medicine, Banja Luka, Bosnia and Herzegovina

**Keywords:** coronary artery disease, bypass graft failure, multispectral imaging, intraoperative imaging, tissue oxygenation

## Abstract

**Significance:**

Coronary artery disease is the leading cause of death worldwide, accounting for 16% of all deaths. A common treatment is coronary artery bypass grafting (CABG), though up to 12% of bypass grafts fail during surgery. Early detection of graft failure by intraoperative graft patency assessment could prevent severe complications.

**Aim:**

We aim to evaluate multispectral imaging (MSI) as a non-invasive, contrast-free method for assessing graft patency during CABG surgery.

**Approach:**

MSI was conducted at video rate during three CABG surgeries and two control surgeries. Two multispectral snapshot cameras captured images in the visible and near-infrared range. Tissue oxygenation and perfusion were derived using linear spectral unmixing and spectral indices.

**Results:**

Significant increases in both oxygenation (12.22±10.24%, p<0.001) and perfusion index (4.50±1.79, p<0.001) were observed after CABG, with no significant changes in control surgeries (oxygenation: −0.36±2.57%, p=0.041; perfusion: 0.41±1.33, p=0.482). These findings demonstrate the ability of MSI to indicate graft patency, in which the bypass graft restores oxygen-rich blood flow.

**Conclusions:**

MSI could offer a valuable tool for surgeons, helping to reduce the risk of graft failure and improve patient outcomes.

## 1 Introduction

Coronary artery disease (CAD) is often caused by one or multiple blockages in the coronary arteries, reducing the flow of oxygen-rich blood to the heart. The gold standard treatment is coronary artery bypass grafting (CABG), involving the placement of a new route, or “bypass,” around blocked arteries to improve blood flow. To create the bypass, healthy blood vessels are grafted from other body parts. However, early graft failure occurs in up to 8% to 12%, often due to technical issues such as improper graft positioning, kinking, or poor distal runoff.^1^ This can lead to perioperative myocardial infarction complicated with arrhythmias, low cardiac output syndrome, and even death.^2^ Timely detection of graft failure during surgery would allow revision, effectively preventing these postoperative complications and improving patient outcomes. However, surgeons face challenges in visually detecting bypass graft failure, necessitating reliable intraoperative assessment methods. Current methods are either invasive, limited in real-time applicability, or require contrast agents, which pose additional risks.

Currently, the gold standard intraoperative method is invasive coronary angiography (ICA). Due to the invasiveness of this catheter-based contrast method, alternatives have been explored, as listed in the comprehensive review by Matiullah et al.^2^ Fluorescence imaging using indocyanine green (ICG) dye shows promising results but requires the injection of the dye.^3^^–^^5^ A more practical and noninvasive method is the transit time flow measurement (TTFM), which is usually combined with high-frequency epicardial echocardiography (HEE).^6^^–^^9^ This allows for quantitative blood flow measurements of the graft, measuring parameters such as graft flow, pulsatility index, and diastolic filling, which can serve as criteria for revision.^10^ However, some clinical studies indicate low patency sensitivity and the determination of appropriate cut-off values of the abovementioned revision criteria seems to differ over surgical scenarios.^11^^,^^12^ Measurement of graft diameter is possible with Doppler ultrasonography, which is shown to be correlated with ICA.^13^ However, the method requires contact and sensitivity also remains low.^14^

To the best of our knowledge, no study has yet evaluated spectral imaging for the purpose of intraoperative graft patency assessment. Spectral imaging has proven effective in reliably measuring ischemic properties of tissue in various surgical scenarios, such as gastrointestinal surgery,^15^^,^^16^ nephrectomy,^17^^,^^18^ and brain surgery.^19^^,^^20^ It benefits from its non-invasive and contrast-free imaging method, where imaging times are short and limit additional surgery time. In addition, it provides direct quantitative 2D maps of physiological parameters, leading to more spatial information on graft patency compared with probe-based methods such as TTFM. In addition, it is capable of providing real-time information on tissue oxygenation and perfusion, whereas many current methods are limited to blood flow dynamics.

In this study, two multispectral snapshot cameras are used to image the exposed heart during open heart on-pump CABG surgery. These do not require spatial or spectral scanning such as traditional systems, as they feature a spectrally resolved detector array. A mosaic filter pattern is deposited directly on the image sensor, with each element of the pattern filtering a specific wavelength of light. As a result, all spectral information is acquired in a single acquisition or “shot.” The spectral datacube is constructed after a demosaicing operation during post-processing.

**Fig. 1:**
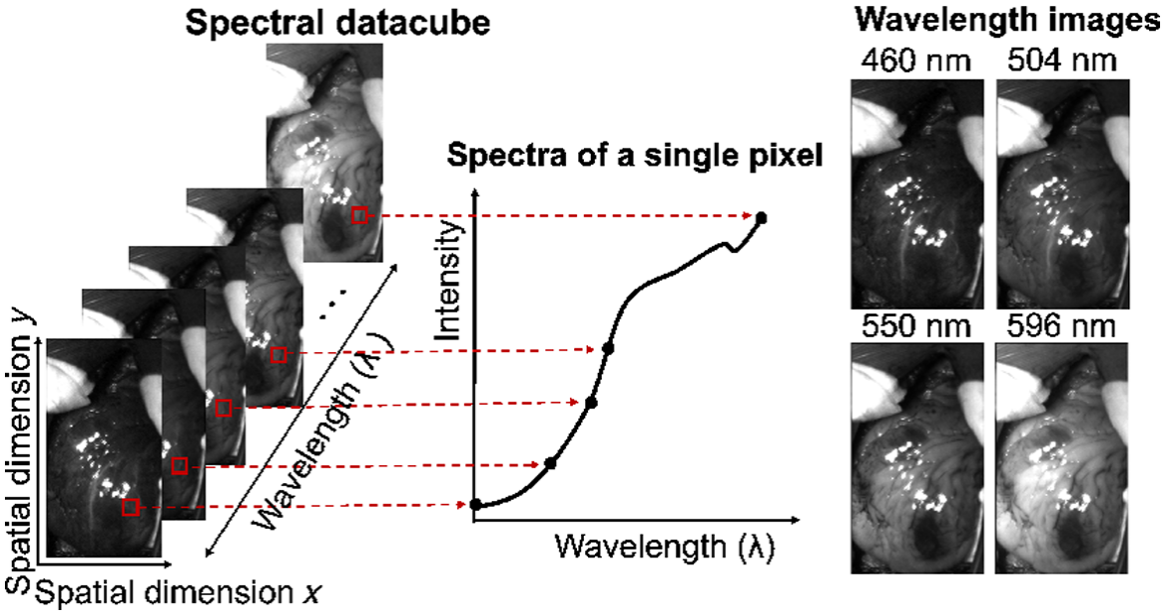
Multispectral imaging acquires a 2D image at different wavelengths. As such, each pixel of the spectral datacube contains a spectrum.

This enables real-time acquisition of spectral data providing direct feedback on tissue parameters. A drawback of these cameras is the reduced spatial and spectral resolution. However, similar to previous work, spectral information from a limited set of wavelengths can be sufficient to reliably derive information on tissue oxygenation and perfusion.^21^^,^^22^ A schematic of multispectral imaging (MSI) is provided in Fig. 1.

MSI was performed intraoperatively and at video rate in five human patients during open heart surgery. Imaging was conducted both before and after surgery, allowing for the study of spectral changes and changes in derived oxygenation and perfusion of the coronary artery.

## 2 Methodology

### 2.1 Surgical Procedure

This study included three patients scheduled for elective open heart on-pump CABG. In all cases, at least one severe stenosis (>70%) in the left anterior descending artery (LAD) was detected during a preoperative ICA. The first patient (CABG 1) had single vessel disease, proximal LAD stenosis of 80%, and a nonsignificant lesion in the distal part estimated as 40% stenosis. Preoperative echocardiography revealed severe aortic stenosis with a mean pressure gradient of 45 mmHg, aortic valve area of $0.8\;{\mathrm{cm}}^{2}$, and a preserved ejection fraction of left ventricle (EFLV) of 60%. The left internal thoracic artery (LITA) was harvested *in situ* in skeletonized fashion using a harmonic scalpel. During cardiopulmonary bypass, the aortic valve was replaced with biological valve St. Jude Epic No 25 and LITA was anastomosed to the mid-segment of the LAD. The second patient (CABG 2) had significant three-vessel CAD. Preoperative echocardiography revealed mild mitral regurgitation and reduced EFLV of 44%. In addition, patient history revealed paroxysmal atrial fibrillation. LITA was harvested in the same fashion, and the great saphenous vein was harvested from both legs in length of 20 cm each. The patient underwent triple bypass surgery: LITA to LAD and two venous aorto-coronary bypasses to obtuse marginal branch (OM) and right coronary artery (RCA) with ligation of the left atrial appendage. The third patient (CABG 3) had multi-vessel disease with hemodynamically significant lesions in mid-RCA, proximal and mid-LAD, diagonal (D), and OM branch. Preoperatively, EFLV was estimated at 66% and all other echocardiographic parameters were within the reference range. Both internal thoracic arteries were prepared *in situ* as previously described. In addition, the great saphenous vein was harvested, measuring 20 cm in length. LITA to mid-LAD anastomosis was constructed in termino-lateral fashion and LITA to diagonal branch in latero-lateral fashion. The right internal thoracic artery was sutured to the distal RCA, and a vein graft was used to bypass the OM branch.

The control group consisted of two patients scheduled for elective open heart on-pump surgery around the same period as the CABG patients but without the presence of CAD. The first patient (control 1) had a degenerative aneurysm of the ascending aorta with a diameter of 5.7 cm. On the preoperative echocardiogram, mild aortic regurgitation was observed with a measured pressure half-time of 400 ms, along with preserved EFLV. Other parameters were within reference values. Replacement of the ascending aorta was performed with preservation of the aortic root. The second patient (control 2) was diagnosed with a 15-cm secundum-type atrial septal defect (ASD) with a left-to-right shunt. The preoperative echocardiogram also showed mild mitral and aortic regurgitation, along with moderate-to-severe tricuspid regurgitation. The right ventricular systolic pressure was measured at 55 mmHg, and both ventricles had preserved contractility. ASD was closed with direct suture, and semicircular annuloplasty of the tricuspid valve by De Vega was performed.

In all patients, crystalloid St. Thomas II cardioplegia was used in amounts ranging from 0.5 to 1 l. All interventions proceeded well without perioperative infarctions or other complications. In addition, all patients were successfully discharged home between the sixth and ninth postoperative day. Approval was obtained from the ethics committee of the Institute of Cardiovascular Diseases of Vojvodina (No: 2594-1/3), and informed consent was collected from all patients. Table 1 shows patient demographics and surgical outcomes.

**Table 1:** Patient demographics, surgical procedure, and outcome (M = male, F = female, C = Caucasian).

	Surgery	Sex	Age	Race	Smoker	Outcome
CABG 1	Myocardial revascularization: LITA-LAD aortic valve replacement	M	78	C	No	+
CABG 2	Myocardial revascularization: LITA-LAD, CxOM, RCA	M	71	C	No	+
CABG 3	Myocardial revascularization: RITA-RCA, LITA-LAD, D, CxOM	F	71	C	Yes	+
Control 1	Ascending aorta repair	M	70	C	Yes	+
Control 2	Atrial septal defect suture	F	58	C	No	+
	Tricuspid valve annuloplasty					

### 2.2 Multispectral Imaging

Images of the exposed heart were acquired using the XIMEA SNAPSHOT VIS (referred to as the *VIS* camera hereafter), a multispectral snapshot camera operating in the visible range (imec, Leuven). The VIS camera captured images at 16 different wavelengths within the 460 to 600 nm spectral range. More specifically, at wavelengths (460±9, 469±10, 475±16, 484±10, 496±16, 504±19, 514±15, 526±19, 532±17, 546±15, 550±15, 564±16, 570±13, 581±18, 589±17, 596±16  nm), it is expressed as peak wavelength ± FWHM. In addition, the XIMEA SNAPSHOT NIR (the *NIR* camera) was used to capture 25 different wavelength images in the 660 to 950 nm range at wavelengths (659±6, 668±5, 687±6, 700±7, 711±7, 728±8, 739±6, 752±8, 766±8, 779±7, 788±8, 802±8, 813±8, 826±8, 840±8, 850±10, 862±10, 876±11, 888±14, 896±13, 910±16, 918±16, 928±17, 939±19, 947±17  nm).

The visual range was selected for its high sensitivity to hemoglobin absorption, whereas the near-infrared range allows deeper tissue penetration, potentially capturing perfusion dynamics in subsurface vasculature. Both cameras have a native resolution of 2048×1088  pixels with a pixel pitch of 5.5  μm and were equipped with a 35-mm C VIS-NIR lens (Edmund Optics). A hyperspectral LED light source (EFFI-RING-HSI, Effilux, France) was used to illuminate the heart, providing adequate power in the investigated spectral range.

**Fig. 2:**
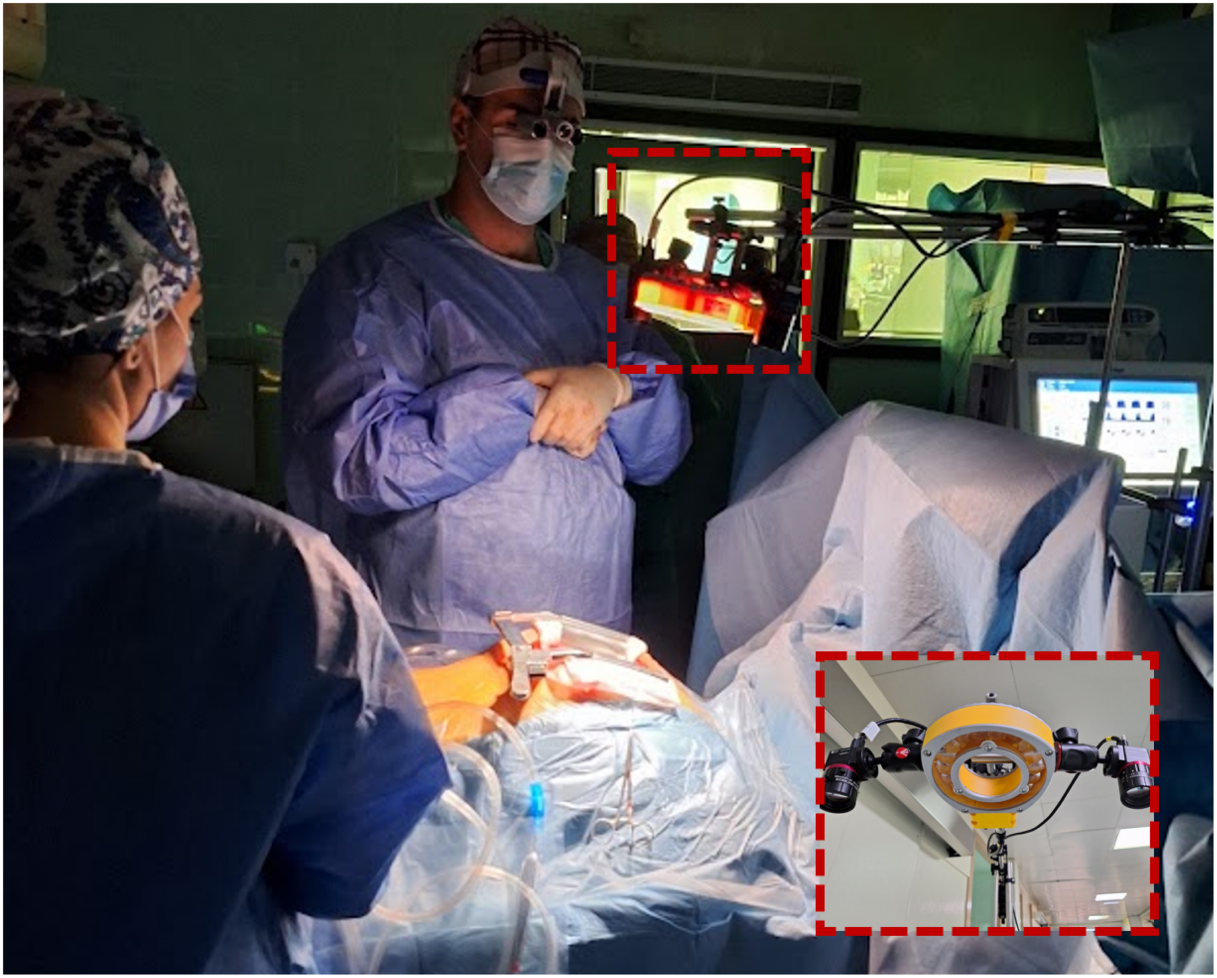
Multispectral imaging system consists of an LED light source and two snapshot cameras. The system is mounted directly above the exposed heart during open surgery.

Imaging was performed twice for each patient: once after exposing the heart, before any other surgical interventions, and again immediately after bypass graft placement, within 10 min after decoupling from cardiopulmonary bypass. One-minute long recordings were taken for both the VIS and NIR camera. To optimize the dynamic range and signal-to-noise ratio (SNR) of the cameras while mitigating motion blur due to the heartbeat, the recordings were acquired at a frame rate between 10 and 20 FPS. Both cameras were used consecutively, with the NIR camera imaging within 5 min after the VIS camera. During acquisition, ambient light was minimized by turning off the operating room lights and closing the blinds. An overview of the imaging setup is shown in Fig. 2.

Due to the set-up, the VIS and NIR camera had a different field-of-view. Pixel-wise registration was not performed because there was no fixed spatial misalignment between acquisitions. In addition, the oxygenation and perfusion parameters described in section are tailored to each camera. However, qualitative comparisons were ensured through manual alignment based on anatomical landmarks.

### 2.3 Image Processing

#### 2.3.1 Radiometric calibration

Radiometric calibration, based on the method described in Ref. 23, was used to convert the raw multispectral images into irradiance values. All operations were implemented in Python 3.11. The same calibration method was used for image data from both cameras. In short, raw images were dark subtracted and demosaicked, and the spectral band images are transferred to a common coordinate system using Lanczos interpolation. To reduce noise while preserving key anatomical features such as edges, the band images were denoised using the bilateral filter from scikit-image.^24^ Sensor-specific spectral imperfections were corrected by applying a correction matrix provided by the camera manufacturer.^25^

For white balancing, a white reference tile (Spectralon 95%, SphereOptics GmbH) was imaged with both cameras. These raw multispectral images were processed using the same radiometric calibration method as described above to convert to irradiance values. The light source spectrum was then constructed by averaging all pixels at each wavelength image and performing min-max normalization. Finally, irradiance data were converted to reflectance data by dividing the calibrated hyperspectral data cubes by the light source spectrum at each pixel. To account for variations in camera-to-heart distance during acquisition, the reflectance data were further L1-normalized and obtained by dividing each reflectance value by the sum of the absolute values of all reflectance values in the spectrum.

#### 2.3.2 Perfusion parameters and RGB reconstruction

Quantitative physiological parameters indicating the oxygenation and perfusion state of the tissue are derived from processed spectral images and compared pre- and post-bypass surgery. These metrics were divided into two categories: those obtained through linear spectral unmixing and those derived from spectral indices.

##### Tissue oxygenation and relative hemoglobin content

Tissue oxygenation and relative hemoglobin content (${\mathrm{Hb}}_{\mathrm{rel}}$), a metric indicative of perfusion, were determined using a linear spectral unmixing algorithm previously published.^21^ Hemoglobin exhibits significantly higher absorption in the visible light spectrum compared with other tissue chromophores such as water and lipids.^26^ As a result, this technique is applied to reflectance data obtained from the VIS camera. Because we specifically evaluate light absorption in the coronary arteries, where hemoglobin is abundant, the contributions of other chromophores are neglected. For each wavelength captured using the VIS camera, the relationship between light absorption and the concentration of oxy- and deoxyhemoglobin is expressed using the Beer-Lambert law as follows:

$$a(w)=-\log(r(w))=(\mu_{a,{\mathrm{HbO}}_{2}}(w)\cdot f_{{\mathrm{HbO}}_{2}}+\mu_{a,\mathrm{Hb}}(w)\cdot f_{\mathrm{Hb}})\cdot L+G,$$ (1)

where $\mu_{a}(w)$ denotes the absorption coefficient at wavelength w in ${\mathrm{cm}}^{-1}$, a(w) and r(w) denotes the L1-normalized absorbance and reflectance at wavelength w, f denotes the volume fraction, L denotes the distance traveled by the photons in cm, Hb denotes hemoglobin, ${\mathrm{HbO}}_{2}$ denotes oxyhemoglobin, and G denotes a constant accounting for losses due to scattering.

Applying Eq. (1) for every wavelength captured using the VIS camera results in a set of 16 equations. After finding the linear least squares solution, values for $f_{{\mathrm{HbO}}_{2}}$, $f_{\mathrm{Hb}}$, and G are obtained. The oxygenation and relative hemoglobin content (${\mathrm{Hb}}_{\mathrm{rel}}$) are then defined as

$$\text{Oxygenation }[\%]=\frac{f_{{\mathrm{HbO}}_{2}}}{f_{{\mathrm{HbO}}_{2}}+f_{\mathrm{Hb}}},$$ (2)

$${\mathrm{Hb}}_{\mathrm{rel}}\;[\mathrm{a.u.}]=(f_{{\mathrm{HbO}}_{2}}+f_{\mathrm{Hb}})\cdot L.$$ (3)

Note from Eq. (3) that ${\mathrm{Hb}}_{\mathrm{rel}}$ is only determined relative to the constant L. The goodness of fit is assessed using $R^{2}$, also known as the coefficient of determination (COD). It is important to acknowledge that the Beer-Lambert law assumes equal path lengths for all wavelengths and that scattering is constant and minimal compared with absorption. However, in reality, red light has a longer path length compared with blue light, and scattering decreases with longer wavelengths.^26^ Nonetheless, these assumptions are considered valid for the investigated spectral range.

##### Superficial and deep perfusion

For each camera, a spectral index was derived to indicate the perfusion state of the tissue. These indices are based on those used in a commercially available clinical hyperspectral system (TIVITA, Diaspective Vision GmbH). Because the wavelengths captured by the TIVITA system differ from those of the VIS and NIR cameras, the indices are adjusted to match as closely as possible.^27^ Specifically, we adapt their proposed *Tissue Hemoglobin Index* to the VIS camera’s spectral range by replacing the isobestic point around 797 nm with that around 500 nm. Their *NIR Perfusion Index* is adapted to the NIR camera by selecting the closest spectral range possible. The VIS camera captures visible light, which has limited tissue penetration, and thus, the derived index is termed superficial perfusion. Conversely, the NIR camera captures longer wavelengths, providing greater tissue penetration, and its corresponding index is defined as deep perfusion.

$$\text{Superficial perfusion }[\mathrm{a.u.}]=\frac{{\overset{¯}{A}}_{530:580\;\mathrm{nm}}}{{\overset{¯}{A}}_{496:504\;\mathrm{nm}}},$$ (4)

$$\text{Deep perfusion }[\mathrm{a.u.}]=\frac{{\overset{¯}{A}}_{826:928\;\mathrm{nm}}}{{\overset{¯}{A}}_{668:739\;\mathrm{nm}}},$$ (5)

where $\overset{¯}{A}$ denotes the mean L1-normalized absorbance in the given spectral range.

##### RGB reconstruction

To provide a reference image familiar to the surgeon, a synthetic RGB image is reconstructed from the spectral image. This facilitates the localization of key structures on the heart, such as arteries and veins. Here, band images of the VIS camera are assigned as color channels of an RGB image (red channel = 597 nm, green channel = 551 nm, and blue channel = 460 nm). To enhance brightness and contrast, histogram equalization is applied.

#### 2.3.3 Assessment of graft patency

**Fig. 3:**
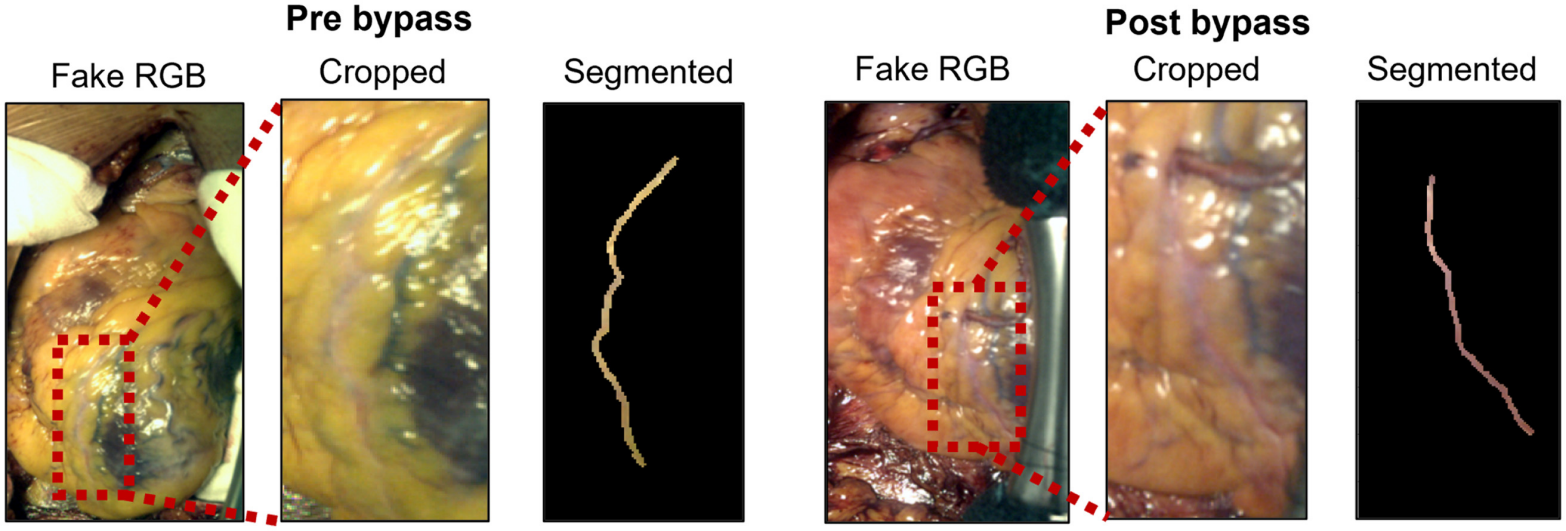
Images are cropped so the field-of-view encompasses the LAD. Oxygenation and perfusion values are then mapped for qualitative analysis. In addition, the artery is manually segmented for quantitative analysis.

To assess the patency of the bypass graft placed in the left anterior descending artery (LAD), images are cropped to encompass the LAD. Subsequently, 2D maps of all physiological parameters are computed, visually emphasizing the differences in the LAD before and after bypass placement. The surgeon then manually segments the LAD based on visual assessment. Segmentations for the VIS camera are performed using the synthetic RGB image, whereas a grayscale wavelength image showing maximal contrast is used for the NIR camera. Pixels with a COD<0.7 are excluded from analysis, as these correspond to specular reflections or areas where light absorption by hemoglobin is not dominant. Pre- and post-surgery images are also ensured to match over the cardiac cycle. Figure 3 illustrates the segmentation procedure for the VIS camera, both pre- and post-bypass.

The mean changes in physiological parameters are computed for all CABG and control surgeries. Statistical significance is tested using linear mixed models (LMMs), with the placement of the bypass as a categorical fixed effect and patient ID as a random effect. LMMs are preferred over standard non-parametric tests because they can deal with the high inter-patient variability of the perfusion parameters with the random effect. To ensure robustness despite the small sample size, statistical significance is set at p<0.01. A p-value less than 0.01 for the model coefficient of the fixed effect indicates a significant difference between pre- and post-surgery. However, due to the limited patient dataset, the confidence in the interpretation of these statistical results should be considered low, and the results should be interpreted as indicative trends.

## 3 Results

### 3.1 Spectral Changes in the Left Anterior Descending Artery Post-Surgery

**Fig. 4:**
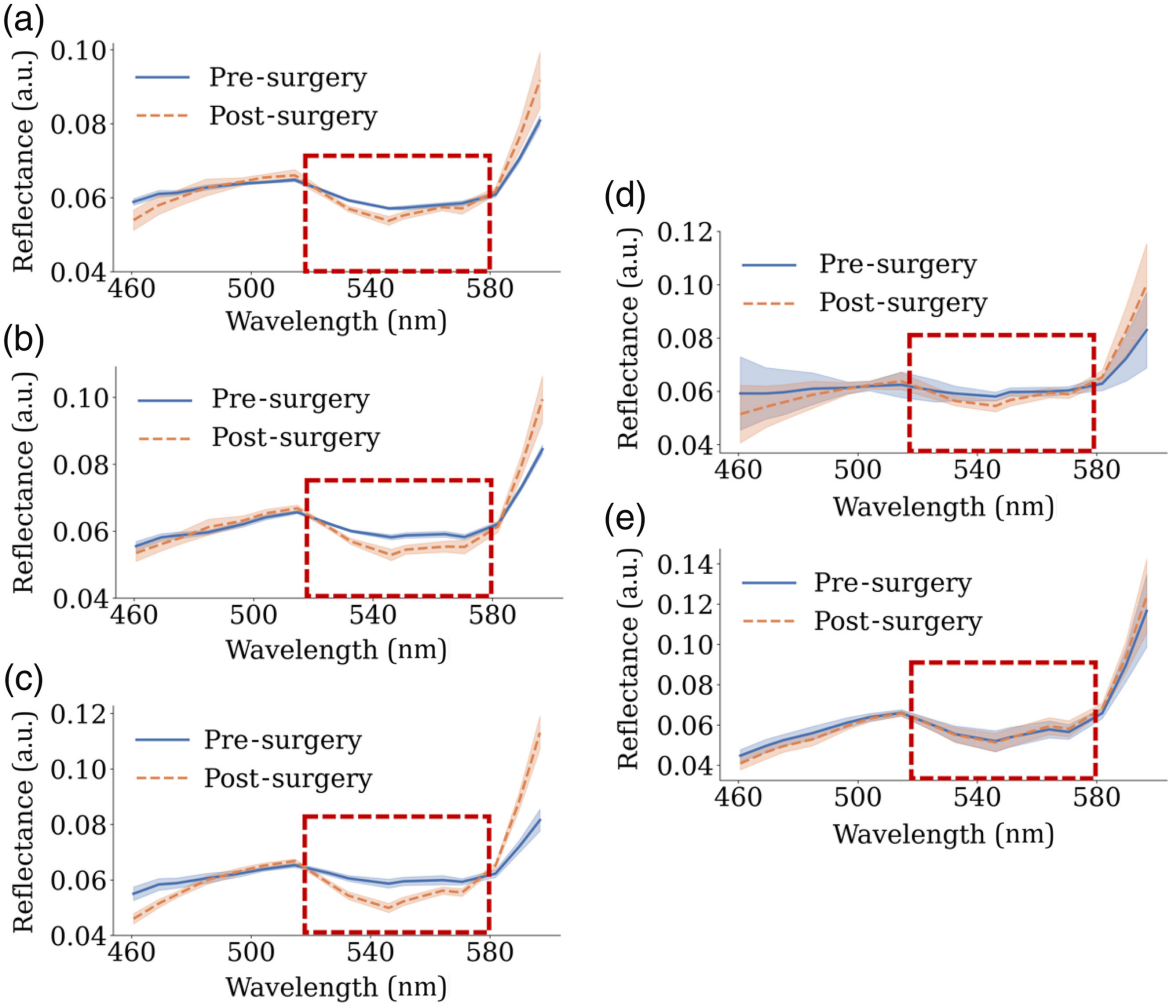
Mean L1-normalized reflectance spectra of the LAD pre- and post-surgery captured with the VIS camera. The shaded bands represent the standard deviation. Spectral differences are observed in the 520 to 580 nm range after bypass placement for CABG 1 (a), CABG 2 (b), and CABG 3 (c), whereas no differences are observed following the control surgeries: control 1 (d) and control 2 (e).

Figure 4 illustrates the mean L1-normalized reflectance spectra from the VIS camera, obtained by averaging the reflectance values of all pixels within the LAD before and after bypass surgery.

In the 520 to 580 nm spectral range, a consistently lower reflectance is observed post-bypass placement in the CABG cases, whereas no significant differences are evident in the control cases. The most pronounced spectral changes occur in CABG 3, where more distinct spectral features are observed in this range following the bypass placement. The mean L1-normalized near-infrared spectra derived from the NIR camera are presented in Fig. S1 in the Supplementary Material. In contrast to the VIS spectra, these NIR spectra exhibit no distinctive changes post-surgery and demonstrate a higher standard deviation.

### 3.2 Oxygenation and Perfusion Changes in the Left Anterior Descending Artery Post-Surgery

**Fig. 5:**
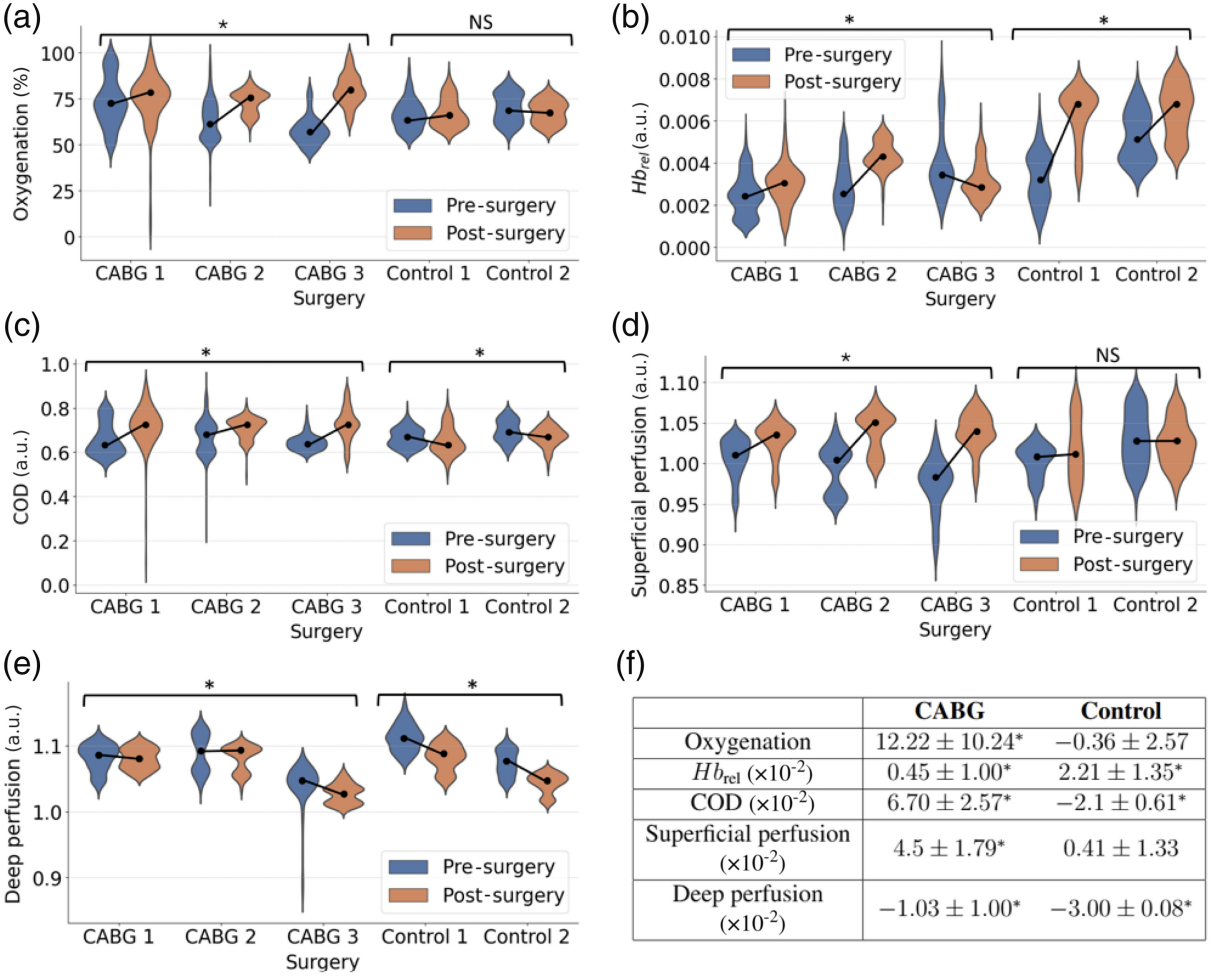
Quantitative differences in perfusion parameters in the LAD pre- and post-surgery illustrated using violin plots. Oxygenation (a) and superficial perfusion (d) exhibit a significant increase in CABG patients, whereas no significant changes are observed in control patients. Differences in ${\mathrm{Hb}}_{\mathrm{rel}}$ (b), COD (c), and deep perfusion (e) are significant in both cases. Mean changes pre-surgery versus post-surgery are shown in panel (f). Statistical significance is indicated by *, and NS denotes not significant.

Violin plots of the estimated physiological parameters in the LAD before and after surgery are presented in Fig. 5. In addition, the mean changes and standard deviations are summarized in Fig. 5(f). Notably, oxygenation and superficial perfusion show significant increases of (12.22±10.24%) and (4.5±1.79%), respectively, following bypass placement in CABG surgeries, whereas no significant changes are observed in control surgeries. The LMM for oxygenation reveals a larger coefficient for the random effect (patient ID) compared with the fixed effect (pre- or post-surgery). This indicates high inter-patient variability in oxygenation levels, which is also evident from different baseline levels in Fig. 5. Pre-surgery oxygenation levels are 73.37±13.33% in CABG 1 and 56.54±6.11% in CABG 3. By contrast, no such variability is observed among the two control patients. For other parameters, the fixed effect outweighs the random effect, suggesting lower inter-patient variability.

**Fig. 6:**
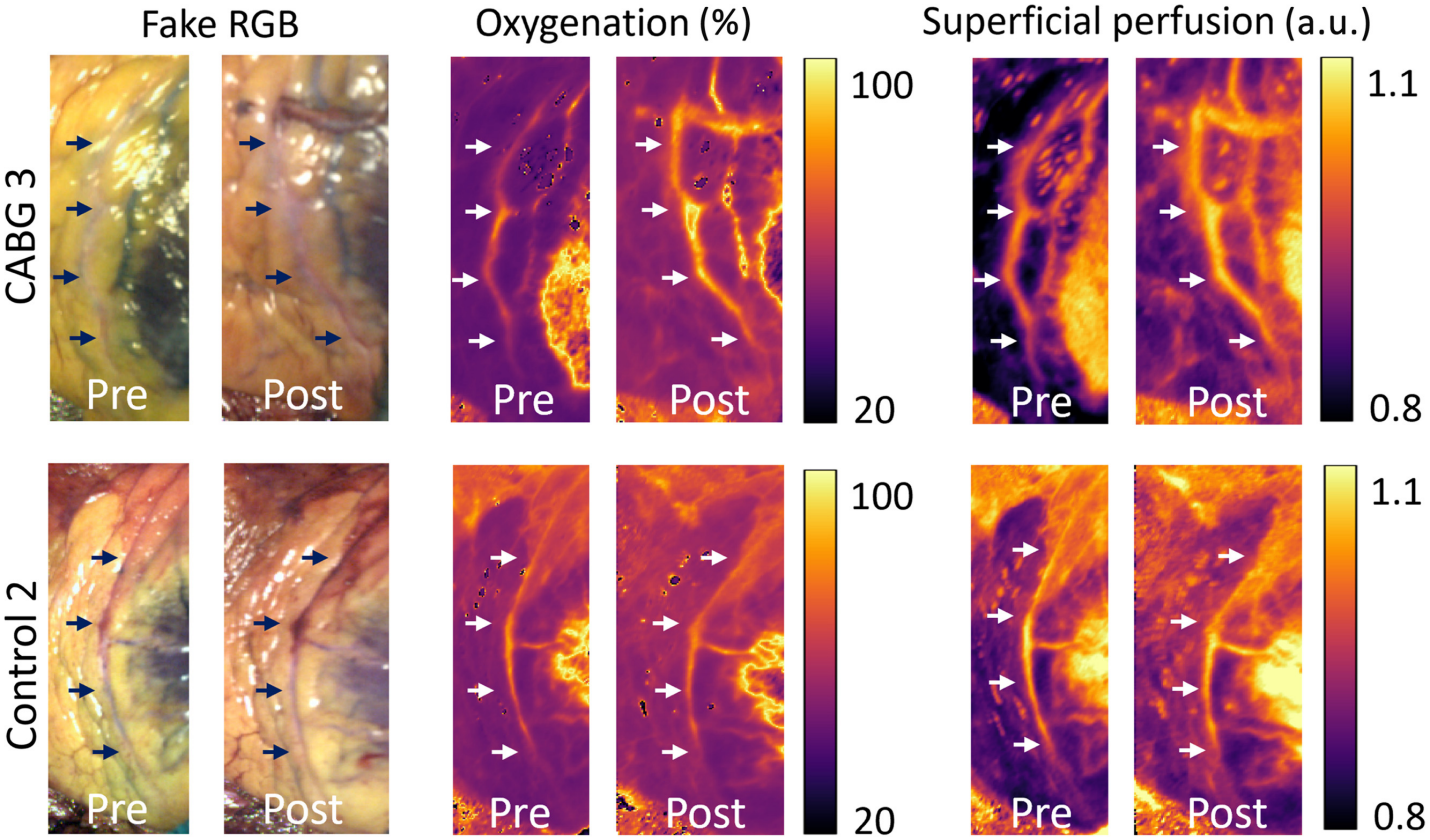
An increase in oxygenation and superficial perfusion can be seen in the LAD pre-surgery versus post-surgery in a CABG case (top), where no evident changes are seen in the control case (bottom). The arrows indicate the location of the LAD.

Relative hemoglobin content (${\mathrm{Hb}}_{\mathrm{rel}}$) increases in both CABG and control surgeries, with a larger increase in the control cases. However, the high standard deviation of these changes indicates low confidence. A significant increase of $(6.70\pm 2.57)\cdot 10^{\left(-2\right)}$ is observed in the COD during CABG surgeries, also reflecting low inter-patient variability and standard deviation. Conversely, the change of $(-2.1\pm 0.61)\cdot 10^{\left(-2\right)}$ observed during control surgeries is not significant. The COD increase suggests that hemoglobin absorption becomes more dominant, resulting in an improved fit of the model described in Eq. (1). Finally, deep perfusion derived from the NIR camera does not exhibit a consistent trend during CABG surgeries, with changes showing high standard deviations. Oxygenation and superficial perfusion display the most distinctive increase after bypass placement during CABG 3.

Figure 6 illustrates 2D maps of these parameters pre- and post-surgery, compared with control 2. In CABG 3, the bypass produces a marked increase in oxygenation and superficial perfusion in the LAD. Conversely, these parameters remain unchanged during the control surgery. Similar parameter maps, including deep perfusion, are provided for other patients in Fig. S2 in the Supplementary Material.

## 4 Discussion

This clinical demonstration of intraoperative MSI during CABG surgery highlights its potential for real-time assessment of graft patency. Spectral differences observed in the 520 to 580 nm region, corresponding to the absorption peaks of oxyhemoglobin (${\mathrm{Hb}}_{O2}$) and deoxyhemoglobin (Hb),^26^ align with literature findings.^21^^,^^28^^,^^29^ As shown in Fig. 4, spectra from post-bypass placement display more distinct features, including a double-dip structure indicative of the ${\mathrm{Hb}}_{O2}$ double absorption peak when transformed to absorbance. These spectral changes are directly linked to the significant oxygenation increases shown in Fig. 5(a).

As illustrated in Fig. S2 in the Supplementary Material, no spectral differences were observed in the NIR measurements, likely due to the lower SNR. Hemoglobin absorption is lower in the near-infrared range, complicating oxygenation and perfusion detection. In addition, the LED ring illumination intensity drops to 30% beyond 850 nm, reducing spectral quality. Combined with the need for high camera frame rates to compensate for motion artifacts, this limitation resulted in reduced SNR. Future iterations could enhance signal quality through improved illumination design or noise reduction techniques.

Oxygenation and superficial perfusion emerged as the most promising parameters for graft patency assessment, both derived from the VIS camera. The spectral unmixing method used to derive oxygenation, along with the superficial perfusion index provided by the commercial HSI system, is well-documented in the literature. This reinforces MSI’s potential as a platform technology for tissue ischemia assessment across various applications.^27^^,^^30^^,^^31^ Figure 6 illustrates clear post-bypass increases in oxygenation and superficial perfusion, enabling straightforward monitoring of graft patency. Furthermore, the COD, which increases consistently post-bypass, could serve as an additional parameter due to its low inter-patient variability. While showing MSI’s potential, these results are mostly indicative due to the small sample size. To increase confidence, a p-value of 0.01 was selected, resulting in no significant changes in oxygenation during the control surgeries. However, it is important to note that this change would have been considered significant with the more commonly used p-value of 0.05.

Deep perfusion, derived from the NIR camera, does not appear suitable for graft patency assessment under the current setup. However, it clearly visualizes coronary arteries and side branches, likely due to the NIR camera’s increased light penetration depth. This capability may attract interest for mapping blood vessels and assessing blood flow in side branches post-surgery.

Although gold standard ICA provides high-resolution anatomical imaging, MSI’s advantage lies in real-time functional assessment without contrast agents. In contrast to TTFM or Doppler ultrasonography, it does not require contact and still provides sufficient spatial information. Future studies comparing MSI-derived parameters with ICA indices are necessary to quantify diagnostic accuracy. With a current processing time of 0.19 s for oxygenation and COD maps using a non-optimized CPU-based Python script and 0.09 s for perfusion maps, real-time assessment is feasible. In addition, the snapshot camera’s C-mount interface facilitates integration with surgical instruments, enabling translation to minimally invasive approaches such as minimally invasive direct CAB (MIDCAB) or totally endoscopic CABG (TECAB) surgery.

This study focused on changes in the LAD to assess graft patency. However, changes in the myocardial tissue could also provide valuable insights. This tissue contains additional chromophores such as myoglobin, lipids, and collagen, making the derivation of oxygenation and perfusion parameters more complex. In addition, myoglobin’s similar absorption spectrum to hemoglobin, and a different oxygen-myoglobin dissociation curve add further challenges.^32^^,^^33^ Future work will adapt analysis methods to address this.

## 5 Conclusion

This pilot study demonstrates the first clinical evaluation of intraoperative MSI for assessing graft patency during CABG surgeries. The spectral region of 520 to 580 nm was identified as critical for detecting oxygenation and perfusion changes, consistent with the absorption characteristics of oxy- and deoxyhemoglobin. Using a visible-range snapshot camera, significant increases in oxygenation and superficial perfusion were observed in the LAD after bypass placement, whereas no significant changes occurred in control surgeries. These findings highlight MSI’s potential as a non-invasive, contrast-free method for monitoring graft patency.

Although the findings demonstrate MSI’s potential, these should be considered exploratory due to the small sample size. Validation on larger cohorts and comparison with gold standard ICA are necessary to quantify diagnostic accuracy and establish clinical guidelines. Such advancements could improve patient outcomes by enabling early detection and prevention of graft failure following CABG surgery.

## Supplementary Material

10.1117/1.JBO.30.3.036001.s01

## Data Availability

The code and data that supports the findings of this study are available upon reasonable request from the corresponding author. The data are not publicly available due to privacy restrictions.
