# Supplementary material for: Real-time multispectral imaging for intraoperative monitoring of coronary artery bypass graft patency
Source: J Biomed Opt. 2025 Mar 10;30(3):036001. doi: 10.1117/1.JBO.30.3.036001 (PMC11897914; doi:10.1117/1.JBO.30.3.036001)
Supplement: Supplementary file 1 [file JBO_030_036001_SD001.pdf]

Supplementary material

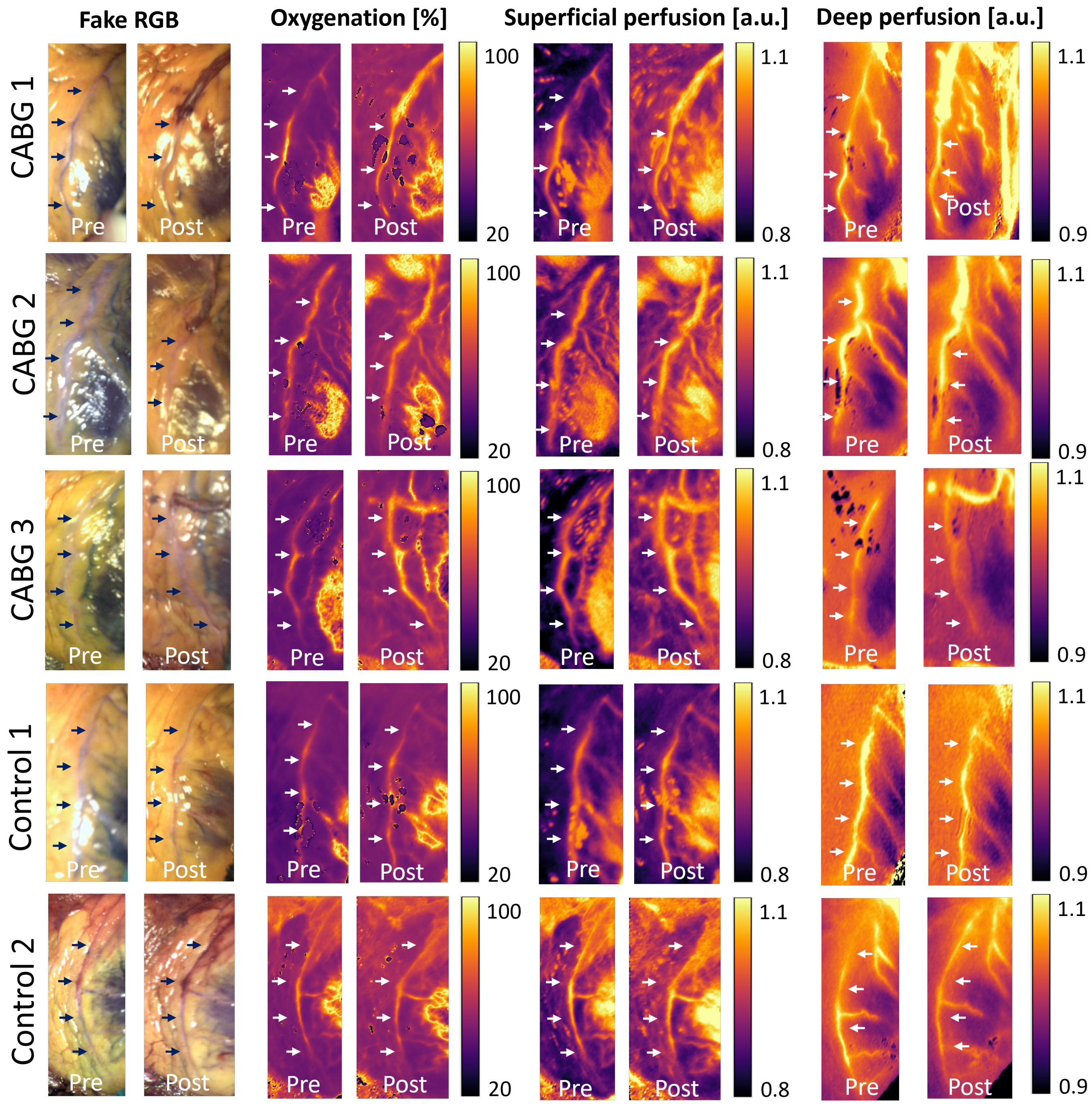

Fig S1 Oxygenation and superficial perfusion increase in the LAD following CABG surgery (top 3 rows), where no changes are observed in the control cases (bottom 2 rows). The deep perfusion index provides a visualization of the LAD and its side branches but shows no significant difference. The arrows indicate the location of the LAD.

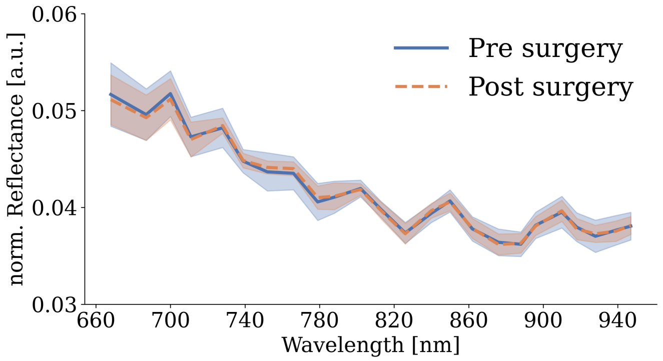

(a) CABG 1

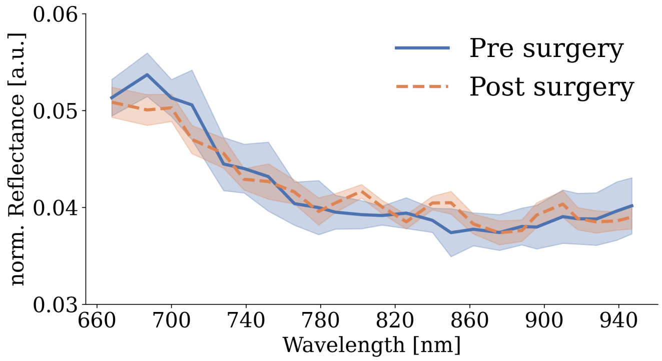

(b) Control 1

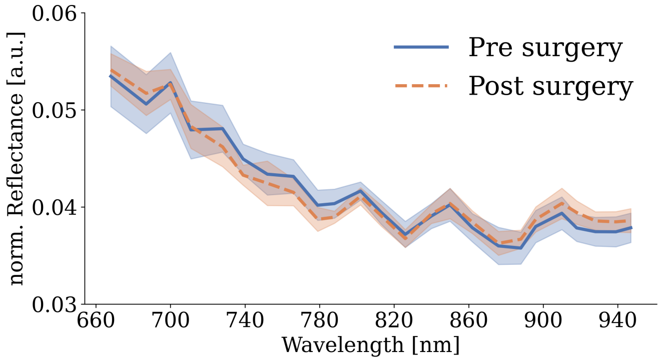

(c) CABG 2

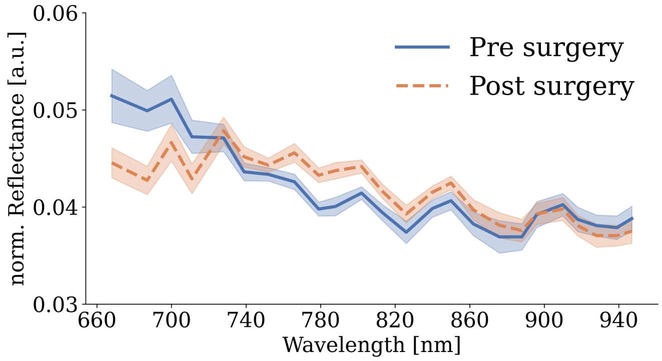

(d) Control 2

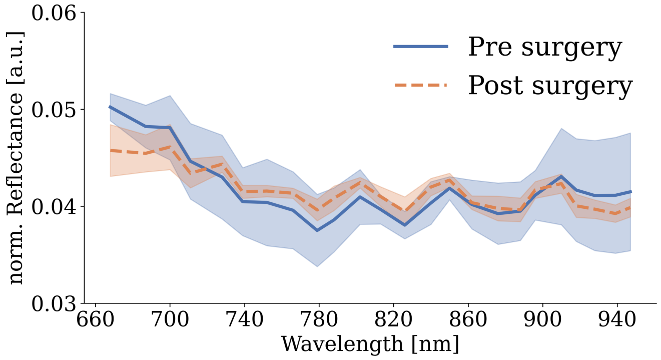

(e) CABG 3

Fig S2 Mean L1-normalized spectra and standard deviation in the LAD pre- and post-surgery using the NIR camera. The spectra display high standard deviation due to the low SNR during acquisition. No distinct spectral differences between the CABG cases and the control can be observed.
